# Coal Tar Antipyretics*Read before the Alumni Association of the Medical College of Indiana, March 31, 1896.

**Published:** 1896-07

**Authors:** J. A. Martin

**Affiliations:** Indianapolis, Ind., Assistant Professor of Chemistry at the Medical College of Indiana


### COAL TAR ANTIPYRETICS.*

By J. A. MARTIN, M.D., Indianapolis, Ind.,
Assistant Professor of Chemistry at the Medical College of Indiana.

The great difference of opinion regarding the virtue of antipyretics of the so-called coal-tar derivation has led me in the past few years to make a close study of this group of medicines.

I shall make it the object of this paper to show the relation existing between the various important members of this group from a chemical, physiological, toxicological, and therapeutic standpoint.

Omitting the discussion of all mechanical mixtures, "anti-this" and "anti-that," which are products of uncertain composition, and are Dr. So-and-So's "favorite prescription," I shall adhere strictly to products of distinct and known chemical composition, such as antipyrin, acetanilid, phenacetin, and lactophenin.

The majority of these products are chemically derived from the first member of the benzene series of hydrocarbons ($C_6H_6$), benzene, through its mono-atomic alcohol or phenol ($C_6H_5OH$), carbolic acid.

Carbolic acid itself acts as an antipyretic in two ways: first by diminishing the production of heat; secondly, by increasing the dissipation or radiation of heat. The exceedingly poisonous and caustic properties of phenol have caused a very limited use of this product as an antipyretic, it having been superseded by its milder and equally efficient derivatives.

The characteristic symptoms of an overdose of carbolic acid are briefly, clonic convulsions, dyspnea, free salivation, lachrymation, conjunctivitis, diminished sensibility, and reduction of temperature. Albuminuria and hematuria are sometimes produced. The *chief* characteristic of the action of phenol is *reduction in arterial pressure.* It paralyzes the vaso-motor centers, and later markedly *depresses the heart,* though death from this drug is usually due to *paralysis of respiration.*

The chemical relation of antipyrin to phenol is shown by its being produced by heating phenyl-hydrazin ($C_6H_5NH, NH_2$) with

*Read before the Alumni Association of the Medical College of Indiana, March 31, 1896.

diacetic ether $\left(\begin{smallmatrix} C_2HO_3 \\ C_2H_2(C_2H_5O) \end{smallmatrix} > O\right)$, when the product is mono-methyl-oxy-quinizine, in which by the replacement of one atom of hydrogen by another methyl group ($CH_3$) di-methyl-oxy-quinizine is formed, which has been given the name of antipyrin on account of its opposition to fever.

Antipyrin was a great improvement as an antipyretic over its predecessor in the coal-tar field, carbolic acid, in that a caustic, poisonous liquid was supplanted by a non-caustic, crystalline powder, soluble in less than its own weight in water, though still retaining many of the physiological and therapeutic properties of phenol.

The great trouble with antipyrin is that it retains *too much* of the *poisonous properties* and *depressing effects* of phenol.

Regarding the nervous system, toxic doses of antipyrin seem to exert a peculiar influence upon the cerebral cortex, as is indicated by the stupor and coma which are noticed. It peculiarly stimulates, then depresses the spinal cord. It is an extreme depressant of the sensory nerve trunks.

Regarding the circulation, antipyrin, in therapeutic doses, increases arterial pressure, while toxic doses lower the pressure. It has been proven to be a *direct paralyzant to the heart of the frog.*

Toxic doses of antipyrin first increase, then markedly *decrease the respiration.*

The common symptoms seen in antipyrin poisoning are ataxia, paraplegia, humid respiration, convulsions with general rigidity, dilated pupils, unconsciousness, and a fall of temperature.

When death occurs, it seems to be due to *failure of respiration.*

The poisoning is due to the action of the decomposition products of the drug upon hemoglobin, to which substance is largely due the condition of cyanosis.

The frequency of an existing idiosyncrasy to antipyrin makes its promiscuous administration very dangerous, since very small doses have produced very alarming conditions.

Acetanilid ($C_6H_5NH,C_2H_3O$) traces its relation to phenol through phenylamine or aniline ($C_6H_5NH_2$), in which one atom of hydrogen is replaced by the acetic acid radical ($C_2H_3O$), acetyl.

It is made by boiling together, for a day or two, equal weights of pure aniline and glacial acetic acid, acetanilid and water resulting. When given in large doses, or to persons having an idiosyncrasy for the drug, acetanilid produces symptoms very similar to those produced by antipyrin, except that the sweating, which does not always occur in either case, is as a rule more profuse following the administration of antipyrin, while the cyanosis is more pronounced following the use of acetanilid.

The latter condition may be explained by the fact that both the decomposition products of acetanilid (aniline and glacial acetic acid) act as deoxidizers upon the blood, both tending to convert the bright-red oxyhemoglobin into the bluish-purple met-hemoglobin, thus preventing the proper oxidation of the tissues.

Usually when acetanilid is given to patients suffering from fever, there is a more decided fall in the *pulse rate* than in the temperature, though both are decreased. Not only the pulse rate, but the size of the pulse is decreased, even to the extent of becoming thready.

This proves acetanilid to be a direct *cardiac depressant.*

Acetanilid produces a fall of temperature by decreasing heat production.

Phenacetin, or acet-para-phenetidin ($C_6H_4<^{OC_2H_5}_{NH(C_2H_3O)}$), is derived from para-phenetidin ($C_6H_4<^{OC_2H_5}_{NH_2}$), which is the ethyl ether of amido-phenol; this later is derived from mono-nitro-phenol ($C_6H_4<^{OH}_{NO_2}$), it in turn being derived from phenol ($C_6H_5OH$), or carbolic acid.

Phenacetin acts as an antipyretic by lessening heat production, through an influence on the nervous system, and without alteration of blood pressure.

Though the blood pressure is not affected by phenacetin, not infrequently a condition of cyanosis is observed where large doses of this drug have been administered. This action is undoubtedly due to the action of one of its decomposition products, probably acetic acid, upon hemoglobin, as in the case of antipyrin and acetanilid poisoning.

The only unpleasant symptoms usually seen following the use of phenacetin are, too profuse sweating, slight ptyalism, slight weakness, dizziness, and dyspnea. In rare cases phenacetin is found to produce collapse, marked cyanosis, great dyspnea and restlessness, cold perspiration and dilated pupils; but these symptoms are always followed by recovery.

Lactophenin ($C_6H_4<^{OC_2H_5}_{NH(C_3H_5O_2)}$), or lactyl-para-phenetidin is related to phenol in exactly the same way as phenacetin, being derived from para-phenetidin.

Lactophenin differs from phenacetin merely in the acid radical; *i. e.*, lactophenin is produced by the replacement of one hydrogen atom in para-phenetidin by the lactid acid radical ($C_3H_5O_2$), lactyl; while in phenacetin the same hydrogen is replaced by the acetic acid radical ($C_2H_3O$), acetyl.

Lactophenin is a white crystalline powder, much resembling in appearance the other members of this group.

Its action though rapid is not abrupt.

The antipyresis produced by lactophenin lasts for from four to six hours, while the subsequent rise of temperature is not accompanied by shivering and other unpleasant effects.

The hypnotic and analgesic effects of this drug are *decidedly more pronounced* than produced by either phenacetin, acetanilid or antipyrin.

Lactophenin, like phenacetin, has no *effect upon respiration or circulation*, but reduces body temperature by lessening heat production, *through an influence upon the nervous system.*

Cyanosis has never been known to follow the administration of lactophenin, since none of its decomposition products have any effects upon hemoglobin.

It seems to me that the keynote was struck when the idea of introducing into para-phenetidin a lactic acid radical, instead of an acetic acid radical, was evolved; thus producing an antipyretic which has no action upon either the respiration or circulation, and at the same time produces no poisonous effects whatever.

In my opinion the day is not far off when antipyrin will be almost wholly discarded, on account of the uncertainty of the degree of its action, and on account of its incompatibility with a large number of drugs in common use; acetanilid will find a home with the surgeon as an antiseptic dressing; and phenacetin, which has done a noble work as a forerunner, will give way to its younger and more worthy brother, *lactophenin.*

